# Supplementary material for: MegaLMM: Mega-scale linear mixed models for genomic predictions with thousands of traits
Source: Genome Biol. 2021 Jul 23;22:213. doi: 10.1186/s13059-021-02416-w (PMC8299638; doi:10.1186/s13059-021-02416-w)
Supplement: Supplementary file 2 — Additional file 2 Review history. [file 13059_2021_2416_MOESM2_ESM.docx]

**1^st^ round**

**Reviewer 1**

Runcie and colleagues present MegaLMM, a new method for fitting multi-variate mixed models, for use in prediction of complex traits (especially animal and plant traits). Unfortunately, I can only understand the basics of the mathematics new method, sorry. However, my feeling is that MegaLMM is a novel and effective tool. The comparisons indicate that for relatively small numbers of individuals, it outperforms existing methods in accuracy, speed and number of traits it can handle. Potentially, the method could become widely used, and to this end, the authors provide an R package (although I have not tested this).

The paper is well-written (Figure 1 is very good) and appears thorough, in particular in the range of alternative methods they compare MegaLMM with, and the three contrasting applications.

Here are a few (semi) major comments

1 - The precision of accuracy estimates (eg Figure 1A) is poor. For example, we can not conclude that MegaLMM produces more accurate models than MCMCglmm, or in fact phenix (note that the datapoints for each method are dependent, so there is little extra support that MegaLMM is top for multiple x-axis values). Why is the precision so poor? You have an abundance of data. If I understand, these are averages over 20 focal traits (so the reported SDs are 4-5 smaller than for individual traits). Further, with 20k traits available, can you not include more to improve the accuracy further?

2 - Similar to 1, I believe you have compared accuracy for only one (grain yield) of the many traits available. By considering more traits, you could increase precision (although here the precision already seems sufficient to discriminate) and avoid accusations that you have "cherry-picked" the trait.

3 - Your speed-up comes from using latent variables to capture the correlation structure. Is there any way to quantify how well these latent variables do? For example, in terms of proportion of correlation they capture? Or by creating a likelihood of the data, given these factors.

Again, apologies for my lack of understanding of the maths. I appreciate that you approximate the full model (1) with a latent factor model (2). When you solve via MCMC, do you actually try to estimate elements of F and Λ?, or only elements of one of these (and integrate across the others)? Thanks

Signed Doug Speed

**Reviewer 2**

Runcie et al. developed a method to fit efficient multivariate linear mixed models using a factor analytic approach and a set of computational and mathematical tricks which increase the sparsity of the factorial decomposition and decrease computational requirements by pre-storing matrix inverses (and discretizing sampling functions) and diagonalizing covariance matrices. This study brilliantly addresses a critical limitation in quantitative genetics, namely the restriction of multivariate analyses to a handful of traits. As such, it could be interesting to scientists in plant and animal breeding, human genetics, and omics data analysis. The manuscript is very well written. The methods are described in great details, which should allow any expert in quantitative genetics to properly replicate the analyses. I only have comments about technical aspects of the study.

The study describes very relevant aspects of the method: computational efficiency, and prediction accuracy in validations schemes where a target trait of interest is masked in testing sets. However, the authors could have included other evaluations that would be very interesting and pertinent to an audience in plant breeding or quantitative genetics.

(1) How do estimated covariance values compare between MegaLMM and other classical approaches like MTG2? Including a figure to show the concordance in estimates of trait covariance between methods could be useful (with few enough traits so that all methods can be applied in reasonable time). Simulations could also be useful to study the accuracy of the estimation, but this type of analyses might be outside the scope of this study.

(2) How robust is MegaLMM to the pre-specified number of latent factors? In practice, the number of latent factors is an important parameter. Is it possible to show to what extent the model's accuracy is sensitive to k? A figure showing the accuracy of parameter estimation and prediction as a function of k could be useful to support the author's claim that "the actual value of k tends to be unimportant as long as it is large enough such that all important factors can be included".

(3) How does MegaLMM compare to other methods, like GBLUP or GBLUP+H when all phenotypic traits are masked in the testing set? The authors report substantial improvements in prediction accuracy by their method, but it is unclear whether this gain is due to the modelling approach (the dissection of genetic and environmental effects, the factorial decomposition among phenotypes) or whether it is due to the use of information at non-target traits in the testing set. For example, does MegaLMM-GBLUP perform better than GBLUP+H because of the modelling approach, or because S at testing lines was incorporated for prediction by MegaLMM-GBLUP. Could you apply MegaLMM-GBLUP in the same validation scheme as GBLUP+H? This type of validations would be all the more interesting that they are practically relevant (when a breeder uses only genotypic data to make predictions, rather than genotypic data + secondary traits).

Minor comments:

Equation (2): Could you explain why X_1B_1 is used only on residuals from factor analysis (and not on factors)?

ll. 474-475: Here, it is unclear how MegaLMM "obviates the need to pre-specify the number of latent factors, making it easier to use." Could you provide a short explanation?

SI, p. 2: The index for each latent factor should be a different letter than k since k denotes the total number of latent factors (similarly to j denoting one of the t traits). For example, in sentences like "regularization on all coefficients for the (k+1)-th latent trait is done relative to the k-th", there is confusion about k as index and k as number of latent factors.

SI, p. 3: Please mention that iG refers to "inverse Gamma".

SI, p. 5: How were {tau_b2R}_j and {phi_b2R}_lj sampled? You did mention, in the section "Fixed Effects", that B_2R follows a horseshoe prior and that you "apply independent global shrinkage parameters for each trait j", but it is still unclear how shrinkage parameters tau_b2R were sampled, exactly.

SI, p. 5: Please mention what V(h^2) refers to.

SI, p. 5: I apologize if I missed something obvious, but I could not recognize the usual definition of the conditional mean for mu (even though Sigma did make sense, through the Woodbury identity). How did you derive the conditional mean for F?

**Reviewer 3**

Summary

MegaLMM is a method for multi-trait genetic predictions or imputation. The model has a Bayesian hierarchical formulation and model parameters are estimated by MCMC.

A n-by-t matrix Y with n observation units having measurements on t traits is modeled with a multivariate linear mixed model. Given posterior draws from the model parameters and random effects for complete observation units, posterior predictions for missing values of a particular phenotype or column or Y are made.

The method is compared to other multivariate mixed models, including MCMCglmm, MTG2, and phenix. In an analysis with 665 observation units and imputation of a gene expression value with 50% missing given 4 "secondary traits" or more completely observed expression values, prediction accuracy improved for MegaLMM and phenix until 512 secondary traits were included. Phenix does not work with more traits than observations, so further traits could not be included for that comparison, but MegaLMM was able to run and make predictions with up to 20842 secondary traits. The comparison methods' running times scale worse than MegaLMM: They are faster with fewer than 32 secondary traits, or fewer than 512 traits for phenix, but have worse trends, such that MTG2 and MCMCglmm were much slower than MegaLMM at 64 secondary traits and unusable under test conditions with more secondary traits.

MegaLMM appears to be a valuable contribution to the field, allowing efficient predictions or imputation of traits with estimates of the genetic contributions to traits that leverage information from very large numbers of genetically correlated traits.

Innovation

In MegaLMM, the Matrix-Normal covariances of Y are modeled as the sum of the contributions from a small number of "latent factors" to each trait plus fixed effects and random effects that are independent across traits.

Y = F Lambda + fixed effects + random genetic effects that are independent across traits + random environmental/noise effects that are independent across traits.

With a low but "sufficiently large" rank estimate of (F Lambda) computation is simplified because condition on F and Lambda, the columns of Y are independent. This allows the improved scalability relative to the comparison methods.

Questions

(1) Page 4 left, line 43. "Finally, to demonstrate the flexibility of MegaLMM, we replaced 256 42432 the genomic relationship matrix with a regression on markers, using a horseshoe prior on the marker coefficients. " In reading this I'm missing a transition -- how does this regression replace a genomic relationship matrix?

(2) In the section on potential extensions, GLMMs with general link functions are mentioned. Another direction to consider would be to keep the linear link but allow non-gaussian errors, say a mixture of Gaussians for a spike-slab prior on genetic effect sizes.

(3) In figure 2.a, prediction performance improved until the number of traits was about the same as the number of observations (around 512 traits for 665 observation units). What were the values of k -- the number of latent factors -- across these runs? Higher for larger numbers of traits? How does prediction accuracy vary when k and t (number of traits) are both varied, or if, say k is fixed at <= 8 or <=32 but t is increased all the way to 20842?

(4) If k, the number of latent factors or rank of (F Lambda) is not sufficiently large, the implied model-based genetic correlations will be mis-estimated. A table or plot showing changes in the predictive performance as k takes values above or below the default could be informative.

(5) If this method hinges on the computational efficiency but statistical adequacy of the factor model or low-rank representation of cross-trait correlations, what can you say about the design of experiments? Should applied researchers emphasize collection of lots of related traits to accurately estimate a few factors or emphasize measurement of genetically divergent (low genetic correlation) traits so that additional traits they may want to predict are in the span of the factor model?

**Author response**

Reviewer #1: Runcie and colleagues present MegaLMM, a new method for fitting multivariate mixed models, for use in prediction of complex traits (especially animal and plant traits). Unfortunately, I can only understand the basics of the mathematics new method, sorry. However, my feeling is that MegaLMM is a novel and effective tool. The comparisons indicate that for relatively small numbers of individuals, it outperforms existing methods in accuracy, speed and number of traits it can handle. Potentially, the method could become widely used, and to this end, the authors provide an R package (although I have not tested this).

The paper is well-written (Figure 1 is very good) and appears thorough, in particular in the range of alternative methods they compare MegaLMM with, and the three contrasting applications.

Here are a few (semi) major comments

1 - The precision of accuracy estimates (eg Figure 1A) is poor. For example, we can not conclude that MegaLMM produces more accurate models than MCMCglmm, or in fact phenix (note that the datapoints for each method are dependent, so there is little extra support that MegaLMM is top for multiple x-axis values). Why is the precision so poor? You have an abundance of data. If I understand, these are averages over 20 focal traits (so the reported SDs are 4-5 smaller than for individual traits). Further, with 20k traits available, can you not include more to improve the accuracy further?

>> The reason that the accuracy estimates in Figure 1A are so uncertain is that the prediction accuracy varies a lot among the gene expression traits in this dataset. For some genes all methods predict with high accuracy, for others all methods fail to predict with any accuracy at all. So the uncertainty here is biological in origin. To show this more clearly, we have included supplemental figures S1 and S2 that break down the results per gene (i.e., per target trait) showing the heterogeneity among genes in how well we can predict. From these figures it’s clear that the ability to predict is correlated across methods and across datasets (i.e., #s of secondary genes), but MegaLMM is almost always at or near the top in accuracy, while MTG2 and sometimes phenix lab behind. The comparison with phenix is interesting as it seems to do as well or better than MegaLMM for genes that we can predict well, but worse for genes that we can’t predict well. It would be interesting to investigate this further but we haven’t done so.

>> While it would be possible to repeat this analysis with more genes to increase the confidence in the comparisons among methods, this would be computationally very costly. In this application we’re predicting a single gene at a time using a set of 4- 20,000 other genes. So a new run of all models is required for each additional gene, and even for MegaLMM this takes many hours when 20,000 other genes are included. It’s true that with MegaLMM we could predict hundreds of genes simultaneously using the additional genes, but we couldn’t do this with the other methods and so it wouldn’t be a fair comparison.

2 - Similar to 1, I believe you have compared accuracy for only one (grain yield) of the many traits available. By considering more traits, you could increase precision (although here the precision already seems sufficient to discriminate) and avoid accusations that you have "cherry-picked" the trait.

>> Thank you for this suggestion. We only compared accuracy for grain yield in this experiment because it is the only “interesting” trait available. The published dataset only includes grain yields and the hyperspectral wavelengths for each line. We don’t see a potential application for predicting hyperspectral wavelengths so didn’t study our ability to do this. Instead, to demonstrate that our results are robust in this application, we repeated the analyses on 19 other similar experiments (other yields and other field treatments) reported in the same paper. In the original submission, we had analyzed the single treatment:year that had the greatest amount of hyperspectral data. For the revised manuscript, in Supplemental Figure S5, we now show that MegaLMM’s performance relative to the competing methods is maintained in nearly all of the experiments.

3 - Your speed-up comes from using latent variables to capture the correlation structure. Is there any way to quantify how well these latent variables do? For example, in terms of proportion of correlation they capture? Or by creating a likelihood of the data, given these factors.

>> Thank you for this suggestion. Unfortunately, we have not found a way to do this that is robust and we are confident in. In our model, all correlation is described by the latent variables, so we can’t measure the proportion directly. In real data, we do not know the true correlations so we can’t directly compare to that, and estimated correlations are noisy (that’s why we use the model).

>> Instead, we have attempted to demonstrate the accuracy of the model through its ability to predict in a cross-validation context where we do know the true result (or at least can model it independently from the model). If MegaLMM were not accurate in estimating correlations, it would not be more successful than univariate models that ignore the multiple traits. Our results clearly show benefits of multi-trait prediction which implies that our estimates of correlation are accurate enough to be useful.

>> In the revised manuscript, we have also included a new simulation study where we generate data with known genetic and residual correlation structures and demonstrate that MegaLMM is at least as accurate at estimating these correlations as any of the competing methods (Figure S3).

>> Going forward, we expect that a better understanding of what these latent variables are actually capturing would be interesting, both as a window into biological mechanisms and potentially as a way to tune the algorithm. However, we think that a full treatment of this topic is complicated and prefer to explore this more in an additional paper. We did discuss interpretability in our previous paper on multi-level sparse factor models (Runcie and Mukherjee 2013) using simulations and we expect similar results to hold here.

Again, apologies for my lack of understanding of the maths. I appreciate that you approximate the full model (1) with a latent factor model (2). When you solve via MCMC, do you actually try to estimate elements of F and Λ?, or only elements of one of these (and integrate across the others)? Thanks

>> Yes, we do get estimates of F and Lambda. Both variables are identifiable in the model because of the heavy-tailed prior on elements of Lambda. We believe that in some cases it may be possible to try to interpret these parameters biologically as we did in our previous paper but have not focused on that here.

Reviewer #2: Runcie et al. developed a method to fit efficient multivariate linear mixed models using a factor analytic approach and a set of computational and mathematical tricks which increase the sparsity of the factorial decomposition and decrease computational requirements by pre-storing matrix inverses (and discretizing sampling functions) and diagonalizing covariance matrices. This study brilliantly addresses a critical limitation in quantitative genetics, namely the restriction of multivariate analyses to a handful of traits. As such, it could be interesting to scientists in plant and animal breeding, human genetics, and omics data analysis. The manuscript is very well written. The methods are described in great details, which should allow any expert in quantitative genetics to properly replicate the analyses. I only have comments about technical aspects of the study.

The study describes very relevant aspects of the method: computational efficiency, and prediction accuracy in validations schemes where a target trait of interest is masked in testing sets. However, the authors could have included other evaluations that would be very interesting and pertinent to an audience in plant breeding or quantitative genetics.

(1) How do estimated covariance values compare between MegaLMM and other classical approaches like MTG2? Including a figure to show the concordance in estimates of trait covariance between methods could be useful (with few enough traits so that all methods can be applied in reasonable time). Simulations could also be useful to study the accuracy of the estimation, but this type of analyses might be outside the scope of this study.

>> We feel that the concordance of estimated covariance values would not be easy to interpret without knowing the true covariances which is impossible in real data. Instead, in the revised version of the manuscript, we added a small simulation study as suggested based on the parameters in the scalability study (the Arabidopsis gene expression dataset). We simulated 128 correlated traits based on artificial G and R matrices and the known genomic relationship matrix among lines. To make the G and R matrices somewhat realistic, we created these based on the empirical covariances of 128 randomly selected genes per covariance matrix (a different set of genes for each matrix). We then used each of the 4 methods to estimate both matrices for subsets of the 128 traits and compared the accuracy of each. This simulation was repeated 20 times with different G and R matrices. Results show that MegaLMM is quite accurate at estimating the covariances at least in this simulation, particularly as the number of traits gets larger. See Figure S3.

(2) How robust is MegaLMM to the pre-specified number of latent factors? In practice, the number of latent factors is an important parameter. Is it possible to show to what extent the model's accuracy is sensitive to k? A figure showing the accuracy of parameter estimation and prediction as a function of k could be useful to support the author's claim that "the actual value of k tends to be unimportant as long as it is large enough such that all important factors can be included".

>> Thank you for this suggestion. In the revision, we have attempted to study this question in more detail using the gene expression dataset. For each # of secondary traits we repeated the MegaLMM analysis with 0.5x and 2x the number of factors. Across target genes and #s of secondary genes, increasing the number of factors has a very small effect on accuracy. But in some datasets, reducing the number of factors from what we originally used did decrease the prediction accuracy. We have added a section explaining how larger K is unlikely to increase (or decrease) accuracy, but will affect computational time and so some optimization here can be useful (see lines 183- 192 in tracked changes document) and Figure S4).

(3) How does MegaLMM compare to other methods, like GBLUP or GBLUP+H when all phenotypic traits are masked in the testing set? The authors report substantial improvements in prediction accuracy by their method, but it is unclear whether this gain is due to the modelling approach (the dissection of genetic and environmental effects, the factorial decomposition among phenotypes) or whether it is due to the use of information at non-target traits in the testing set. For example, does MegaLMM-GBLUP perform better than GBLUP+H because of the modelling approach, or because S at testing lines was incorporated for prediction by MegaLMM-GBLUP. Could you apply MegaLMM-GBLUP in the same validation scheme as GBLUP+H? This type of validations would be all the more interesting that they are practically relevant (when a breeder uses only genotypic data to make predictions, rather than genotypic data + secondary traits).

>> This is a very interesting suggestion. We implemented this for the wheat dataset (and the additional wheat datasets mentioned above to Reviewer #1), and consistently find that the entire benefit of MegaLMM in this context is through the use of information from non-target traits in the testing set. We have included this result in Figure 3A and S5, and discussed the implications in lines 256-264 and 473-489 (line numbers refer to the “tracked changes” document. Note that in the application of GBLUP+H, the same phenotypic information from the testing lines is also used, so the original comparison was a fair comparison against HBLUP and GBLUP+H. In both of these models, hyperspectral data from the testing lines is used to predict grain yield of the testing lines. The reviewer is correct that in most breeding applications today, predictions are made only from genotype data and are made before the testing lines are even grown.

However, we aimed to show here that there may (in some cases) be tremendous benefit to incorporating data on the testing lines as they grow too, and MegaLMM is particularly useful in this context.

Minor comments:

Equation (2): Could you explain why X_1B_1 is used only on residuals from factor analysis (and not on factors)?

>> The distinction between the predictions in X_1 and X_2 is that the coefficients of X_1 are given diffuse (flat) priors, while the predictors of X_2 are given informative priors that perform regularization. If we included X_1B_1F as a predictor of the factors, this would not be identifiable with X_1B_1 because of the lack of regularization. B_2F and B_2R are identifiable because a single large value in B_2F could potentially explain the same variation as multiple large values in B_2R (because B_2F is multiplied by Lambda), and so the model will favor allocating variation to B_2F over B_2R if is is consistent with the covariance modeled by Lambda.

ll. 474-475: Here, it is unclear how MegaLMM "obviates the need to pre-specify the number of latent factors, making it easier to use." Could you provide a short explanation?

>> We apologize. This sentence is probably not quite correct. The user does need to pre-specify the number of factors, but we believe that the choice is less critical for prediction accuracy than it is in other factor models, so MegaLMMl is considerably more robust to choice of K than other factor models. We have removed this statement here, leaving the previous discussion of K.

SI, p. 2: The index for each latent factor should be a different letter than k since k denotes the total number of latent factors (similarly to j denoting one of the t traits). For example, in sentences like "regularization on all coefficients for the (k+1)-th latent trait is done relative to the k-th", there is confusion about k as index and k as number of latent factors.

>> Thank you for pointing this out. We have replaced the notation with K (not bold to distinguish it from the kinship matrices discussed) representing the number of latent factors and k being an index.

SI, p. 3: Please mention that iG refers to "inverse Gamma".

>> We have made this addition.

SI, p. 5: How were {tau_b2R}_j and {phi_b2R}_lj sampled? You did mention, in the section "Fixed Effects", that B_2R follows a horseshoe prior and that you "apply independent global shrinkage parameters for each trait j", but it is still unclear how shrinkage parameters tau_b2R were sampled, exactly.

>> The sampling algorithm for these parameters follows after equation S10 in step 4(a) of Section 1.3 on page 7. In this section, we describe sampler steps for a generic univariate linear mixed model. D_\beta is a diagonal matrix with diagonal elements the product of a tau and a phi. The reason that the subscripts are dropped here is that this generic model applies to both columns of F (in which case the diagonal elements are tau_b2F_j * phi_b2F_ij), and columns of Y (in which case some of the diagonal elements are tau_b2R_j * phi_b2R_ij) and others are tau*\phi, corresponding to elements of Lambda). Regardless of the precise identities of these variables, each diagonal element is a product of a phi and tau, and so the sampling strategies are the same. These parameters are given half-Cauchy priors, but we sample by expanding to a mixture of inverse-Gammas so that we can use a Gibbs sampler.

SI, p. 5: Please mention what V(h^2) refers to.

>> Thank you for catching this. This should have been K(h^2) which refers to the prior covariance for the vector u as a function of h2. We have corrected this and included a definition here. The precise form of this function is not critical as long as it returns a positive semi-definite matrix, but generally it returns a block-diagonal matrix with each block a product of an element of h2 and a pre-defined covariance matrix (i.e., kinship matrix for a particular family of random effects).

SI, p. 5: I apologize if I missed something obvious, but I could not recognize the usual definition of the conditional mean for mu (even though Sigma did make sense, through the Woodbury identity). How did you derive the conditional mean for F?

>> We apologize. The notation with all the transposes and complicated indices were difficult here. The model is equivalent to:

Y = Xb + m + e, b~N(mu_b,S_b), e~N(0,S_y) where we assume m is known. So the conditional posterior of b|y,rest is N(mu,Sigma) where:

Sigma = (X S_y^{-1} X + S_b^{-1})^{-1}

Mu = Sigma*(XS_y^{-1}(y-m) + S_b^{-1}*mu_b)

(following the section on conjugate priors here:

https://en.wikipedia.org/wiki/Conjugate_prior#When_likelihood_function_is_a_continuou

s_distribution)

In our case, we substitute:

X -> Lambda

m -> (\tilde{Mt_R)_i)

S_b -> D_f

S_y -> D_Yt_i

mu_b -> \mu_Ft_i

Reviewer #3: Summary

MegaLMM is a method for multi-trait genetic predictions or imputation. The model has a Bayesian hierarchical formulation and model parameters are estimated by MCMC.

A n-by-t matrix Y with n observation units having measurements on t traits is modeled with a multivariate linear mixed model. Given posterior draws from the model parameters and random effects for complete observation units, posterior predictions for missing values of a particular phenotype or column or Y are made.

The method is compared to other multivariate mixed models, including MCMCglmm, MTG2, and phenix. In an analysis with 665 observation units and imputation of a gene expression value with 50% missing given 4 "secondary traits" or more completely observed expression values, prediction accuracy improved for MegaLMM and phenix until 512 secondary traits were included. Phenix does not work with more traits than observations, so further traits could not be included for that comparison, but MegaLMM was able to run and make predictions with up to 20842 secondary traits. The comparison methods' running times scale worse than MegaLMM: They are faster with fewer than 32 secondary traits, or fewer than 512 traits for phenix, but have worse trends, such that MTG2 and MCMCglmm were much slower than MegaLMM at 64 secondary traits and unusable under test conditions with more secondary traits.

MegaLMM appears to be a valuable contribution to the field, allowing efficient predictions or imputation of traits with estimates of the genetic contributions to traits that leverage information from very large numbers of genetically correlated traits.

Innovation

In MegaLMM, the Matrix-Normal covariances of Y are modeled as the sum of the contributions from a small number of "latent factors" to each trait plus fixed effects and random effects that are independent across traits.

Y = F Lambda + fixed effects + random genetic effects that are independent across traits + random environmental/noise effects that are independent across traits.

With a low but "sufficiently large" rank estimate of (F Lambda) computation is simplified because condition on F and Lambda, the columns of Y are independent. This allows the improved scalability relative to the comparison methods.

Questions

(1) Page 4 left, line 43. "Finally, to demonstrate the flexibility of MegaLMM, we replaced 256 42432 the genomic relationship matrix with a regression on markers, using a horseshoe prior on the marker coefficients. " In reading this I'm missing a transition -- how does this regression replace a genomic relationship matrix?

>> It has been shown (e.g., Van Raden 2008) that the GBLUP model is equivalent to a regression on all markers with an iid N(0,\sigma^2) prior on each marker. In fact, many of the commonly used genomic prediction methods such as the Bayesian alphabet family methods can all be written as a model where the genetic values are multivariate normal with a covariance matrix which equals XDXt where D is a weight matrix applied to each marker that often must be learned. Therefore, there is an equivalence between regressions on markers and appropriate genomic relationship matrices. However, we have decided to remove this section from the manuscript because our subsequent work over the past months has shown that efficiently implementing the regression-on-markers approach requires some additional care and method development and we feel that this would be better served with a full treatment in a separate paper. Therefore we have removed this sentence and the corresponding result from Figure 3

(2) In the section on potential extensions, GLMMs with general link functions are mentioned. Another direction to consider would be to keep the linear link but allow non-gaussian errors, say a mixture of Gaussians for a spike-slab prior on genetic effect sizes.

>> Thank you for this suggestion. We are currently working on implementing a spikeslab type prior for the genetic effects, and more flexible residual distributions would definitely be useful for many types of data. Unfortunately, the computational burden in our Gibbs sampler will likely be much greater. We added text about this in the discussion (lins 610-615 of “tracked changes” document).

(3) In figure 2.a, prediction performance improved until the number of traits was about the same as the number of observations (around 512 traits for 665 observation units). What were the values of k -- the number of latent factors -- across these runs? Higher for larger numbers of traits? How does prediction accuracy vary when k and t (number of traits) are both varied, or if, say k is fixed at <= 8 or <=32 but t is increased all the way to 20842?

>> We added labels in Figure 2 showing K for each MegaLMM model. Because of the efficiency of the MegaLMM algorithms, we can use fairly large K without a huge computational burden. The effect of K on model performance is discussed above and in the new Figure S4.

(4) If k, the number of latent factors or rank of (F Lambda) is not sufficiently large, the implied model-based genetic correlations will be mis-estimated. A table or plot showing changes in the predictive performance as k takes values above or below the default could be Informative.

>> See response to Reviewer #2 above and refer to the new Figure S4 and S2.

(5) If this method hinges on the computational efficiency but statistical adequacy of the factor model or low-rank representation of cross-trait correlations, what can you say about the design of experiments? Should applied researchers emphasize collection of lots of related traits to accurately estimate a few factors or emphasize measurement of genetically divergent (low genetic correlation) traits so that additional traits they may want to predict are in the span of the factor model?

>> Thank you for this suggestion. Given the results of the experiment suggested by Reviewer #2, we have added a section to the discussion about when MegaLMM might have the greatest impact for breeding (or prediction in general, lines 473-489). Adding additional highly correlated traits is not likely to provide much benefit per se, while adding un-correlated traits potentially provides new information to be used in prediction. However, pre-selecting which traits to include needs to be done with care to avoid biasing the cross-validation criterion, so we believe that one of the practical benefits of MegaLMM is that it doesn’t hurt it to just throw all traits that you can find into the model at once without having to pre-select.

**2^nd^ round**

**Reviewer 1**

The authors have answered all my original comments, thank you.

Having been unable to understand the paper well for my first review, I have now read again in detail, and I understand it better. This leads me to have few more, minor comments

I believe you do not obtain a standalone prediction model from MegaLMM (as you would from, say, multivariate blup, when feasible). I believe you acknowledge this in Line 135, when you explain how you perform prediction by including missing values in the model. However, I think this point should be included in the limitations in the conclusions (if I am correct, and have not missed it)

Please remove the unnecessary decimals in x the y-axis label for Figure 2B (10,000.000 looks like 10M, etc). Also, the bottom label should be 0.0001 (not 0). So maybe switch to 10^3, 10^-2, 10^-1, etc

Typo at top of page 10? "An important feature of MegaLMM is that *the* multiple random effect terms can be included"

In supplement, I suggest you add eq 1 from main text to start (and maybe also eq 2) so that it becomes self contained. Also, useful to redefine M (=F+t)

Thanks

**Reviewer 2**

The authors have fully and brilliantly addressed all my comments. I appreciate that they added pertinent information about estimation accuracy for covariance parameters (Figure S3), sensitivity to hyperparameter K (Figure S4) and cross-validation in “CV1” (only genomic data in testing set; Figures 3 and S5). I agree with their interpretation of the usefulness and relevance of MegaLMM (i.e., improved accuracy through the observed secondary traits in the testing set).

I have no other comment, except for a typo in “used each to calculate[d] an empirical correlation matrix” in supplementary information (legend of Figure S3).

My sincere congratulations to the authors for an excellent and very useful work.

**Reviewer 3**

The authors have addressed all of my concerns.

**Author response**

Thank you for your comments. We have added a line to the conclusions about using

MegaLMM in a stand-alone context, altered the y-axis of Figure 2B and the color

schemes of all figures, added the preliminary equations to make the supplement selfcontained

and fixed the two noted typos. We have also switched the license to the MIT
